# The Characteristic of Fe as a *β*-Ti Stabilizer in Ti Alloys

**DOI:** 10.3390/ma14247516

**Published:** 2021-12-08

**Authors:** Jin Min, Yanhua Guo, Jingzhe Niu, Juexian Cao, Zhonggang Sun, Hui Chang

**Affiliations:** 1 Tech Institute for Advanced Materials and College of Materials Science and Engineering, Nanjing Tech University, Nanjing 211816, China; 201961203141@njtech.edu.cn (J.M.); sunzgg@njtech.edu.cn (Z.S.); ch2006@njtech.edu.cn (H.C.); 2 State Key Laboratory of Porous Metal Materials, Northwest Institute for Nonferrous Metal Research, Xi’an 710016, China; jniu@c-nin.com; 3 Hunan Institute of Advanced Sensing and Information Technology, Xiangtan University, Xiangtan 411105, China; 4 Shangi Institute for Advanced Materials (Nanjing) Co., Ltd., Nanjing 210038, China

**Keywords:** phase transition, titanium alloys, *β* phase stabilizer, first-principle calculations

## Abstract

It is well known that adding elements, especially *β*-Ti stabilizers, are holding a significant effect on titanium alloy strength due to the solution and precipitate strengthening mechanisms. In order to reveal the Fe strengthening mechanism in titanium, this study investigate the effect of Fe on the stability of *β*-Ti and the phase transition between *α*, *β* and *ω* phase with first-principle calculations. According to our study, Fe is a strong *β*-Ti phase stabilizer could owe to the 3d orbital into e_g_ and t_2g_ states which results in strong hybridization between Fe-*d* orbital and Ti-*d* orbital. The phase transition from *ω* to *β* or from *α* to *β* becomes easier for Fe-doped Ti compared to pure titanium. Based on our results, it is found that one added Fe atom can lead the phase transition (*ω* → *β*) of at least nine titanium atoms, which further proves that Fe has a strong stabilizing effect on *β*-Ti phase. This result provides a solid guide for the future design of high-strength titanium with the addition of Fe.

## 1. Introduction

Titanium alloys are widely used in aerospace, marine engineering, biomedical and other fields owing to their low density, high specific strength, high corrosion resistance and high biocompatibility. Similar to most metal applied in industry, titanium is holding several allotropes which including *α*-Ti (HCP structure), *β*-Ti (BCC structure) and some intermediate phase retained between the *β* → *α* transformation [1]. For the category of titanium type at room temperature, one can classify them into three main type which including *α*, *β* and *α* + *β* type based on their *β* phase composition. Normally, the *α* + *β* type or *β* type titanium alloys are holding some characteristics such as higher strength, lower modulus, better formability, high welding ability, etc. when compared with whole *α* alloy. Therefore, the main role of the alloying elements in titanium alloy is to promote or restrain the formation of *β* phase and thus effect the alloy’s performance [2,3].

Fe has been a popular element used in recent years to design new low elastic modulus and high strength titanium alloys due to its strong *β* phase stabilization and low cost. Our latest work [4,5,6] reveals that Ti-Fe binary alloys with trace Fe addition (Fe < 4 wt.%) are presenting an excellent mechanical property, better corrosion resistance and good biocompatibility when compared with pure titanium and most Ti-X binary alloys designed for dental implants application due to the phase composition controlling and grain refinement effects. By adding 3 wt.% Fe in Ti-25Nb alloy, Lee et al. [7] found that the bending strength/modulus ratio of the Ti-25Nb-3Fe is enhanced about 41.4% when compared to that of Ti-6Al-4V. In addition, when 1 wt.% or more Fe is added to the Ti-25Nb alloy, the typical orthorhombic *α*” and *ω* phases are suppressed and the *β* phase titanium is retained. When the Fe content reaches 9 wt.%, the Ti-11Nb-9Fe alloy exhibits a single *β*-phase microstructure with a decrease in elastic modulus and an enhancement in plastic strain and elastic energy. The microstructure, bending strength, and bending modulus of Ti-5Nb-*x*Fe with Fe content of 1–5 wt.% were studied by Hsu. et al. [8,9]. And it was found that the microstructure and mechanical properties of the alloy were sensitive to the Fe content. When 4 wt.% Fe was added, the *β*-phase with BCC crystal structure was completely retained. Furthermore, it was indicated that the Ti-5Nb-2Fe alloy had the best bending properties. H. Min et al. [10,11] investigated the microstructure, tensile properties, and corrosion behavior of Ti-15Mo-*x*Fe alloys. They found that the development of *α*’’martensite and athermal *ω* phases was greatly suppressed due to Fe addition. The microstructure characterization of Ti-5Cr-*x*Fe [12], Ti-6Al-*x*Fe [13] showed that the Fe content has a strong influence on the phase composition and crystal structure.

Based on the above studies, it can be found that Fe is a promising alloying element and has been widely used in the design of new titanium alloys due to its good *β*-phase stabilization. Nevertheless, the physical origin of its stabilization mechanism is still unclear. Therefore, the density-functional calculations for Fe doped in *α*, *β* and *ω* phase of titanium are carried out in this study. Through the charge density distribution, projected density of states (PDOS), the mechniam of Fe stabilization on *β*-Ti has been studied.

## 2. Computational Details

In our study, the density functional calculations of the generalized gradient approximation (GGA), as implemented in the Vienna ab initio Simulation Package (VASP) [14], are utilized in our study. Perdew-Burke-Eznerhof (PBE) [15] GGA for the exchange-correlation potential is used for all calculations. The all-electron projector augmented plane-wave (PAW) [16] method is adopted. The 3*s*, 3*p*, 3*d*, 4*s* and 4*p* states were selected as reference states for the pseudo-potentials of Ti and Fe atoms. For obtaining accurate total energy and reaction barriers, the plane-wave basis energy cutoff is set to 500 eV. The reciprocal space is performed by the Monkhost-Pack k-point scheme for the Brillouin zone sample, where a 9 × 9 × 9 grid is used for geometric relaxation and 18 × 18 × 24 is used for the static calculation of the density. Meanwhile, the complete geometrical optimization is completed when the Hellmann-Feynman force applied to each atom is less than 10^−3^ eV/Å. After the structural relaxation is completed, high-precision static calculations are performed using the tetrahedral method with Blöchlcorrections [17] and with an energy convergence criterion of 10^−8^ eV.

## 3. Results

Firstly, we perform structure optimization for *α*, *β* and *ω* phase of titanium and determine their lattice constants as shown in Table 1. For comparison, we also show lattice constants obtained from experiments [18,19] and other calculation work [20]. Obviously, our calculated lattice parameters for different phase of titanium agree well with the experimental values and the calculated values. Compared their total energy listed in Table 1, which can be found that the *ω* phase of titanium has the lowest energy at its equilibrium states and the *β* phase is unstable with 113 meV/atom higher in energy. Our calculations are also consistent with most theoretical predication [21,22,23,24].

To illustrate the effect of iron on the stability of the *β*-phase, the total energy, charge density, and projected density of states (PDOS) of the *α*, *β*, and *ω* phases were calculated. It should be noted that there are two possible substitution sites (named as 1a site and 2d site) for Fe doped in *ω* phase. The formation energy for every Fe doped in titanium alloy are calculated as:

E_f_ (Fe, *x*) = E_tot_ (Fe, *x*) − E(Fe) − N × E(Ti, *x*) with *x* = *α*, *β* and *ω* (1)

**Figure 1:**
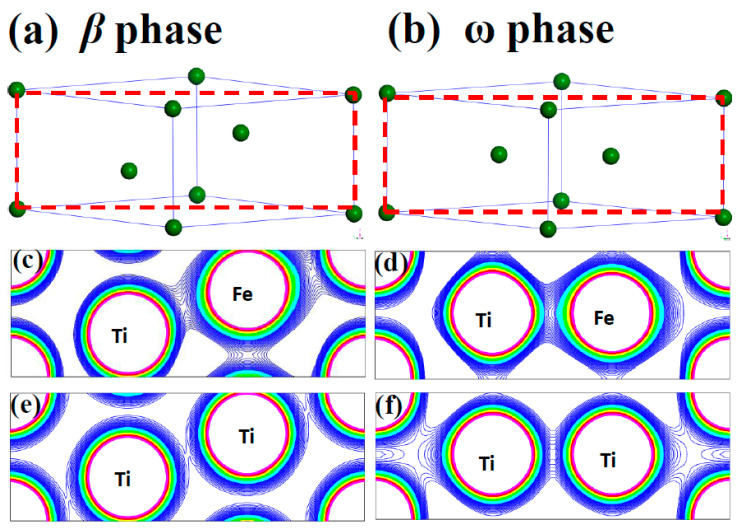
The atomic configurations of (**a**) *β* phase Titanium and (**b**) *ω* phase Titanium. And the Charge density of plane indicated as the dash line rectangle in (**a**) and (**b**) for Fe-doped *β* phase Titanium (**c**); Fe-doped *ω* phase Titanium (**d**); *β* phase Titanium (**e**) and *ω* phase Titanium (**f**).

**Figure 2:**
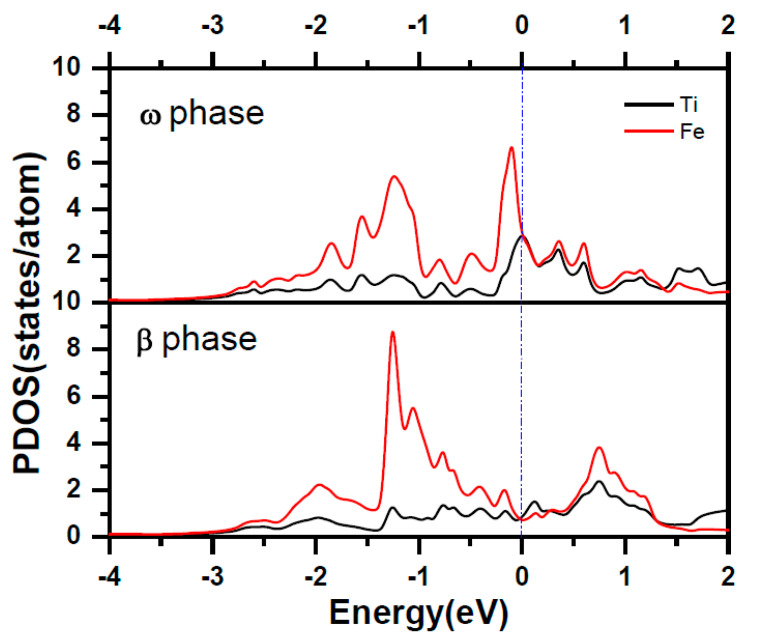
The projected density of states for Ti atom and Fe atom for Fe-doped *ω* phase and *β* phase titanium.

**Figure 3:**
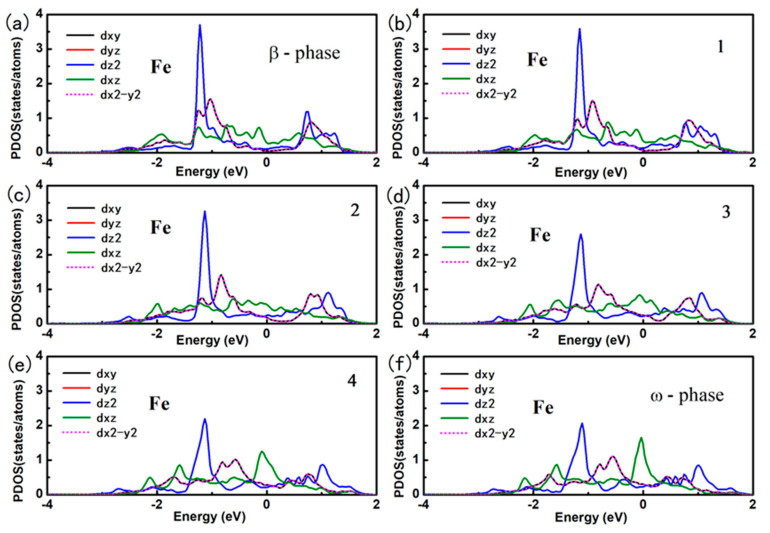
The projected density of states for Fe atom for Fe-doped *ω* phase and *β* phase titanium.

**Figure 4:**
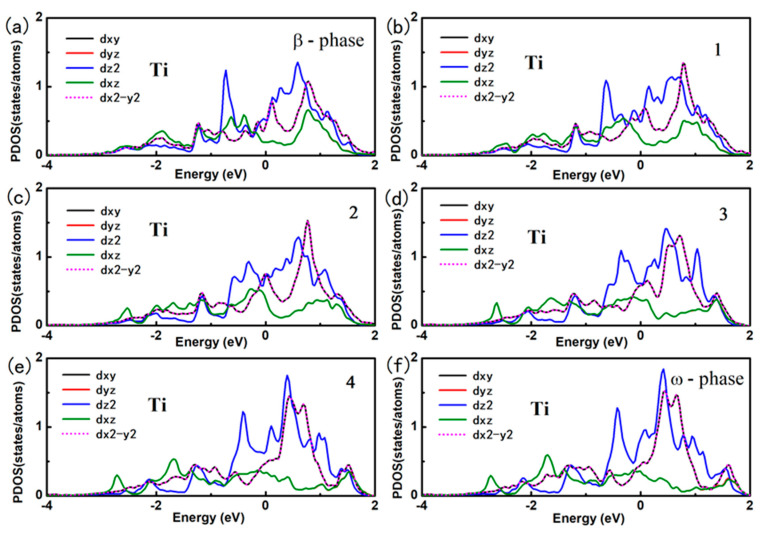
The projected density of states for Ti atom for Fe-doped *ω* phase and *β* phase titanium.

Here, E_tot_ (Fe, *x*) refers to the total energy of Fe doped in *x* phase titanium. E (Ti, *x*) is the total energy per atom with *x* phase. And E (Fe) is energy per atom of Fe in BCC structure. we use the formation energy for per Fe atom to identify the stability of the Fe doping in different phase of titanium alloy. The formation energy for per Fe atom doped in deferent phase of titanium alloys are calculated as: 0.58 eV, −0.03 eV and 0.88 eV for Fe doped in *α*, *β* and *ω* phase titanium, Clearly, the substitution Fe prefers to dope in the *β* phase titanium, indicating that the Fe is a *β* stabilizer element. The contour of the charge density distribution for Fe doped *β* and *ω* phase of titanium are shown in Figure 1, contours start from 2.0 × 10^−5^ e/Å^3^ and increase consecutively by a factor of 1.2. The position of substitution site labeled as Fe are indicated in Figure 1c,d. For comparison, the contour of the charge density distribution for *β* and *ω* phase of titanium are also shown Figure 1e,f. The results show that the charge density and interactions between Ti-Ti in the *β*-phase are much weaker than those in the *ω*-phase The results show that the charge density and interactions between Ti-Ti in the *β*-phase are much weaker than those in the *ω*-phase Therefore, the titanium in the *β*-phase is more unstable than that in the *ω*-phase. Obviously, the charge density between the Ti-Fe is much more than between the Ti-Ti in *β* and *ω* phase titanium. The charge density between Ti-Fe for Fe doped in *β* phase titanium is still a little bit smaller than that for Fe doped in *ω* phase titanium. However, Fe atom has eight nearest neighbor Ti in *β* phase while there is only three nearest neighbor Ti in *ω* phase. This difference results in the stability of Fe doped in *β* phase and *ω* phase titanium. To further investigate the interaction between the Fe and Ti in *β* phase and *ω* phase titanium, we give the atomic projected DOS of Fe and Ti in Figure 2. We draw the partial DOS of four points on the *ω*-*β* transformation path in Figure 3 and Figure 4 when a 2 × 2 × 2 supercells is doped with one Fe atom. There is a resonance peak near the Fermi level for Fe and Ti for Fe doped in *ω* phase titanium while the resonance peak locates at 1.4 eV lower the Fermi Level for Fe doped in *β* phase titanium. The height of the resonance peaks indicates the strength of the hybridization between the Fe atom and Ti atom and the position of the resonance peak refers to the hybridization energy level. The lower energy lever and the stronger hybridization strength means the stronger interaction between atoms. From the PDOS shown in Figure 2, Figure 3 and Figure 4, we conclude that the interaction between Fe and Ti in *β* phase is much stronger than that in *ω* phase, which is responsible for the lower energy for Fe doped in *β* phase titanium.

**Figure 5:**
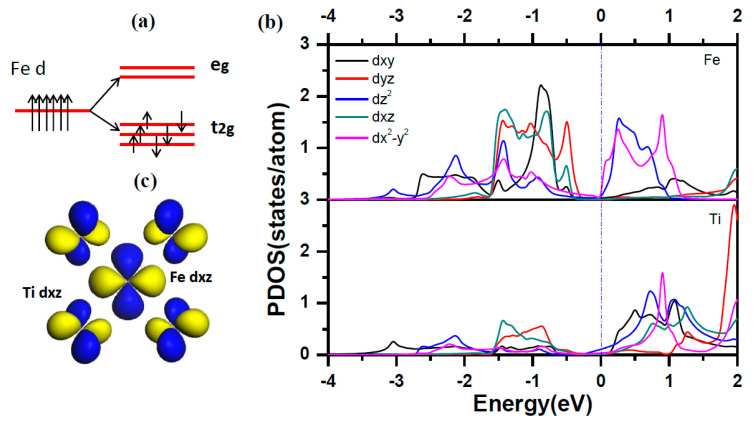
(**a**) The description of d orbitals in octahedral crystalline filed. (**b**) The orbitals projected density of states for Ti and Fe in Fe-doped *β* phase titanium. (**c**) the charge density distribution for description of the interaction for Fe-*dxz* and Ti-*dxz*.

In order to find the physical mechanism for Fe as a *β* phase stabilizer element, we give the orbital projected DOS of Fe and Ti for Fe doped in *β* phase titanium. Under the octahedral crystalline field, the five *3d*-orbital of Fe atom split into two energy levels, namely e_g_ and t_2g_ as shown in Figure 5a. The double degenerated e_g_ states involves *d*x^2^-y^2^ and *d*z^2^ and the triplet degenerated t_2g_ states includes *d*_xy_, *d*_xz_ and *d*_yz_. For Fe atom, the six d electron full filled the lower three t_2g_ states according to Hund rule as the PDOS shown in Figure 5b. The splitting of the 3*d*-orbital of Fe atom leads to the directional feature of orbitals. It leads to largest hybridization between the Fe and the Ti at each octahedral vertex. The PDOS in Figure 5b shows that the two d electron of Ti occupies the *d*_xz_ and *d*_yz_ orbitals which follows the same energy range of the orbitals of Fe atoms. It indicates the strong hybridization between Ti-*d*_yz_/Ti-*d*_xz_ orbitals and Fe-*d*_yz_/Ti-*d*_xz_ orbitals, which is in accordance with the obtained data shown in Figure 2. The Figure 5c shows the charge density distribution for the interaction of Fe-*d*_xz_ and Ti-*d*_xz_.

**Figure 6:**
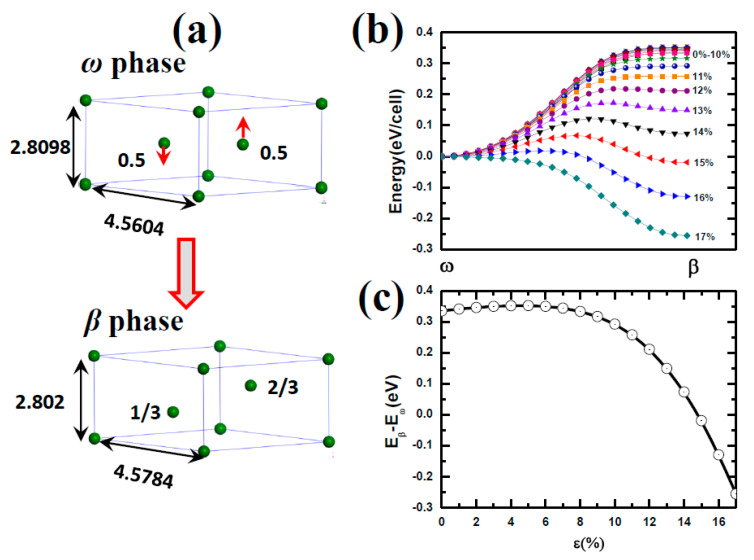
(**a**) The description of the *ω* phase to *β* phase transformation. The red arrow indicates the atomic displacement in the phase transformation. (**b**) The energy barriers for the *ω* phase to *β* phase transformation under different strain. (**c**) The energy difference between the *ω* phase to *β* phase transformation under different strain.

To illuminate the dopant effects on the phase transition between *ω* phase titanium and *β* phase titanium, we calculated the atomic diffusion paths and the energy barrier between *ω* phase and *β* phase. From Figure 6a, one can find the lattice mismatch between *ω* phase and *β* phase is quite small. The large difference between the two structure is that the 2d atoms of *ω* phase titanium locate at (1/3, 2/3, 1/2) and (2/3, 1/3, 1/2) while these two titanium atom move to (1/3, 2/3, 1/3) and (2/3, 1/3, 2/3) in the *β* phase, respectively (see the red arrows shown in Figure 6a). The energy barriers are plotted in Figure 6b. Under the ambient environment, the *ω* phase titanium is very difficultly transformed to *β* phase titanium due to the fact that the *β* phase titanium is about 333 meV/cell higher in energy than the *ω* phase titanium. With the hydrostatic pressure, applied evenly to all parts of the surface of the object. An increase in hydrostatic pressure reduces the volume of the object under pressure, but does not change its shape, this phase transition become easy [25]. As increase of the compressive strain, the energy difference between the *β* phase and the *ω* phase decreases. Especially for *ε* = 15%, the energy difference is almost zero. Further increasing the compressive strain, *ω* phase titanium would be automatically transformed to *β* phase titanium because there is no energy barrier between the transition pathways. The Figure 6c indicated the energy barrier between *β* phase and *ω* phase with various compressive strain. Our results predicted that the *ω* to *β* phase transition appears with 15% compressive strain, consistent with the results for phase transition under hydrostatic pressure [20]. It is impossible to achieve 15% [beyond 150 GPa] smaller volumes in *β*-phase titanium because corresponding pressures would be similar to those in the Earth core [20]. The *β* phase has remained stable up to 87.7 GPa [18] and the observation of the *ω* → *β* transition in the range of 40~80 GPa using angle-dispersive synchrotron X-ray diffraction [20].

**Figure 7:**
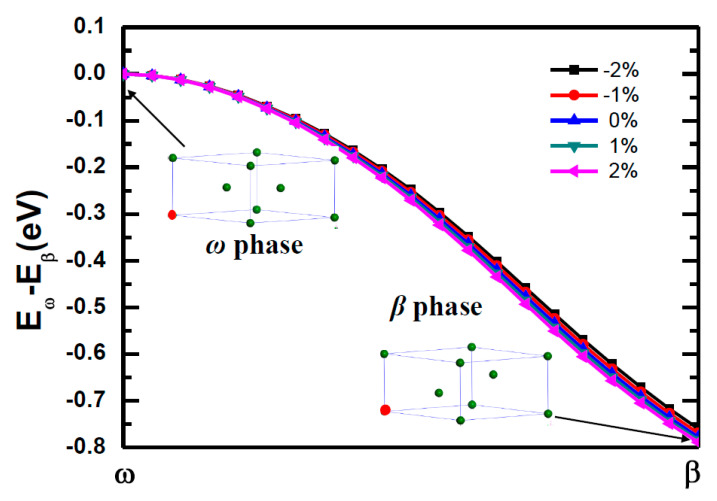
The energy barriers for the Fe-doped *ω* phase to Fe-doped *β* phase transformation under different strain. The inset indicated the atomic configuration of Fe doped *ω* titanium and Fe-doped *β* titanium. The red balls and the green ball refer to the Fe and Ti atom, respectively.

**Figure 8:**
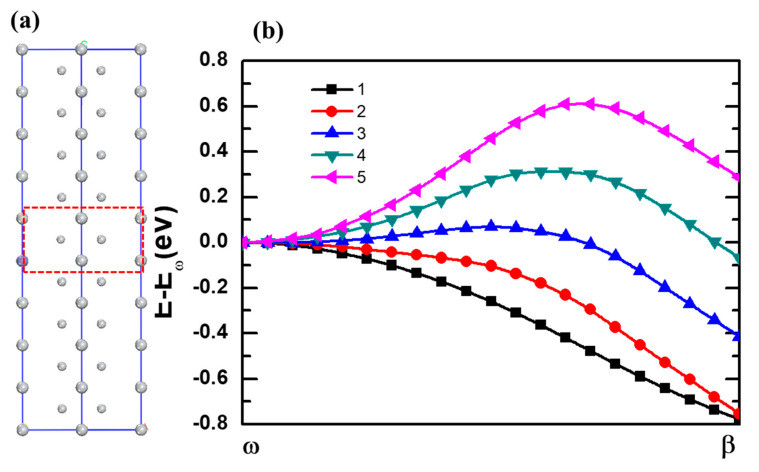
(**a**) The description of Fe doped in 1 × 1 × *n* *ω* phase titanium supercells. (**b**) The energy barriers for the *ω* phase to *β* phase transformation with Fe doped in 1 × 1 × *n* supercells.

**Figure 9:**
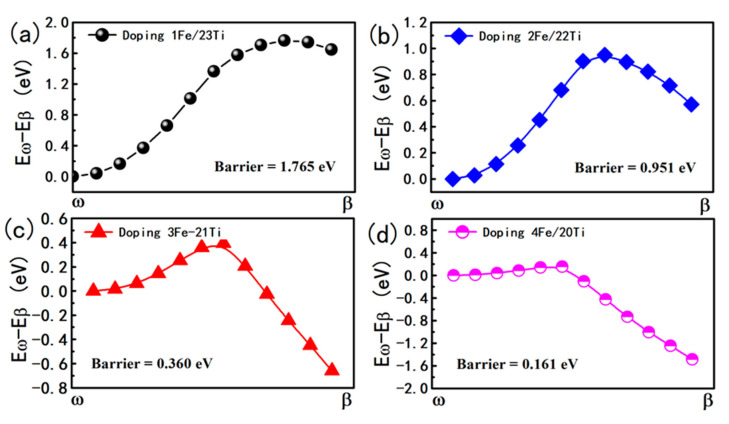
The energy barriers for the Fe-doped *ω* phase to Fe-doped *β* phase transformation under different Fe concentration in 2 × 2 × 2 supercell. (**a**) doping-1Fe in the body center. (**b**) doping-2Fe in the body centers and other in the top corner. (**c**) doping-3Fe all in the face centers. (**d**) doping-4Fe three face center and one body center.

The important finding here is that the *ω* phase titanium spontaneously transform to the *β* phase titanium after Fe doped at 1a site as shown in Figure 7. Our calculations shown that the Fe doped *β* phase titanium is about 0.75 eV/cell lower in energy than the Fe doped *ω* phase titanium. The strain shows negligible influence on *ω* → *β*. If the dopant Fe take the 2d site of the *ω* phase titanium, the *ω* to *β* phase transition will also occur due to the fact that the Fe doped *ω* phase titanium is unstable with 0.72 eV/cell in energy higher than Fe doped *β* phase titanium. From above calculations, one can learn that the dopant Fe have huge potential to induce the *ω* to *β* phase transition. To further qualify the potential of Fe induced phase transition, we perform calculations of *ω* to *β* phase transition for Fe doped 1 × 1 × *n* (*n* = 1−5) supercell of titanium as shown in Figure 8. Figure 8a give a description of one Fe doped in 1 × 1 × *n* (*n* = 1−5) supercell for *ω* phase titanium. Figure 8b give the specific data of energy barrier for *ω* → *β* of each consideration supercells. For *n* = 1−3, one can find that the Fe doped *ω* phase titanium can easily transform to *β* phase transition due to the fact that there are no energy barriers along the transition pathways and the Fe doped *β* phase is lower in energy than Fe doped *ω* phase titanium. Although the Fe doped 1 × 1 × *n* supercell of titanium with *β* phase is lower in energy than that with *ω* phase, the phase transition should overcome an energy barrier of 0.32 eV. The phase transition is difficult to realize for Fe doped 1 × 1 × 5 supercell of titanium due to the two factor: energy barrier of 0.60 eV and the fact that Fe doped *ω* phase titanium is stable in energy than Fe doped *β* phase titanium. Hence, one dopant Fe can induce to the *ω* → *β* transformation of at least nine titanium atoms We also check the supercells of 2 × 2 × 2 as shown in Figure 9, and the result of the calculation is consistent with the result of the unit cell.

## 4. Conclusions

In conclusion, the effect of dopant Fe on the phase stability (*α*, *β* and *ω*) and the phase transition (*ω* → *β*) From their total energy, charge density and DOS, we have demonstrated that Fe is an excellent *β* phase stabilizer. We reveal the physical origin for the dopant Fe as a stabilizing element of *β*-phase because under octahedral crystalline filed the five d orbital of transition metals will split into t_2g_ and e_g_ states which leads to the strong hybridization between Fe-d and Ti-d in *β* phase titanium. The power of the dopant Fe induced *ω* to *β* phase transition is further calculated. Furthermore, it can be determined that one dopant Fe can induce to the *ω* → *β* transformation of at least nine titanium atoms Our calculations give guidance to design high strength titanium-based alloys.

## Figures and Tables

**Table 1:** Calculated equilibrium lattice parameters for *α*, *β* and *ω* phase of titanium and their total energy.

Structure	Space Group		Unit Cell			
				(Å)		
			*a*	*b*	*c*	Energy (eV/Atom)
*α*	*P6_3_/mmc*	This work	2.935	2.935	4.654	−7.890
		Ref. [17]	2.920	2.920	4.717	
		Ref. [18]	2.939	2.939	4.650	
*β*	*Im3m*	This work	3.237	3.237	3.237	−7.783
		Ref. [16]	3.310	3.310	3.310	
		Ref. [18]	3.255	3.255	3.255	
*ω*	*P6/mmm*	This work	4.594	4.594	2.813	−7.896
		Ref. [17]	4.588	4.588	2.837	
		Ref. [18]	4.575	4.575	2.828	

## Data Availability

All data generated or analyzed during this study are included in this published article.
